# P-734. Clinical Characteristics and Outcomes of Respiratory Viral Infections in the Endemic Era: A Comparative Study of COVID-19, Influenza, and RSV

**DOI:** 10.1093/ofid/ofae631.930

**Published:** 2025-01-29

**Authors:** Yu Jung Choi, Joon Young Song, Jang Wook Sohn, Won Suk Choi, Seong-Heon Wie, Jacob Lee, Jin-Soo Lee, Hye Won Jeong, Joong Sik Eom, Eliel Nham, Hye Seong, Jin Gu Yoon, Ji Yun Noh, Hee Jin Cheong, Woo Joo Kim

**Affiliations:** Division of Infectious Diseases, Department of Internal Medicine, Guro Hospital, Korea University College of Medicine, Seoul, South Korea, Seoul, Seoul-t'ukpyolsi, Republic of Korea; Division of Infectious Diseases, Department of Internal Medicine, Korea University College of Medicine, Seoul, South Korea, Seoul, Seoul-t'ukpyolsi, Republic of Korea; Division of Infectious Diseases, Department of Internal Medicine, Korea University College of Medicine, Seoul, Korea, Seoul, Seoul-t'ukpyolsi, Republic of Korea; Korea University Ansan Hospital, Ansansi, Kyonggi-do, Republic of Korea; Division of Infectious Diseases, Department of Internal Medicine, St. Vincent Hospital, College of Medicine, The Catholic University of Korea, Seoul, Korea, Kyonggi-do, Kyonggi-do, Republic of Korea; Division of Infectious Diseases, Department of Internal Medicine, Kangnam Sacred Heart Hospital, Hallym University College of Medicine, Seoul, South Korea, Seoul, Seoul-t'ukpyolsi, Republic of Korea; Division of Infectious Diseases, Department of Internal Medicine, Inha University School of Medicine, Incheon, South Korea, Incheon, Inch'on-jikhalsi, Republic of Korea; Department of Internal Medicine, Chungbuk National University Hospital, Cheongju, Republic of Korea / Department of Internal Medicine, Chungbuk National University College of Medicine, Cheongju, Republic of Korea,, Cheongju, Ch'ungch'ong-bukto, Republic of Korea; Division of Infectious Diseases, Department of Internal Medicine, Gil Medical Center, Gachon University College of Medicine, Incheon, Korea, Incheon, Inch'on-jikhalsi, Republic of Korea; Division of Infectious Diseases, Department of Internal Medicine, Guro Hospital, Korea University College of Medicine, Seoul, Korea, Seoul, Seoul-t'ukpyolsi, Republic of Korea; Division of Infectious Diseases, Department of Internal Medicine, Korea University College of Medicine, Seoul, South Korea, Seoul, Seoul-t'ukpyolsi, Republic of Korea; Division of Infectious Diseases, Department of Internal Medicine, Korea University College of Medicine, Seoul, South Korea, Seoul, Seoul-t'ukpyolsi, Republic of Korea; Division of Infectious Diseases, Department of Internal Medicine, Guro Hospital, Korea University College of Medicine, Seoul, South Korea, Seoul, Seoul-t'ukpyolsi, Republic of Korea; Division of Infectious Diseases, Department of Internal Medicine, Korea University College of Medicine, Seoul, South Korea, Seoul, Seoul-t'ukpyolsi, Republic of Korea; Division of Infectious Diseases, Department of Internal Medicine, Korea University College of Medicine, Seoul, South Korea, Seoul, Seoul-t'ukpyolsi, Republic of Korea

## Abstract

**Background:**

On May 5, 2023, the World Health Organization declared the end of the public health emergency for COVID-19, marking the transition into the endemic era. In the endemic situation, there is limited data on the epidemiological of respiratory virus infections. This study aimed to compare the epidemiology and clinical outcomes of three representative respiratory viral infections (COVID-19, influenza and RSV).

**Table 1.**
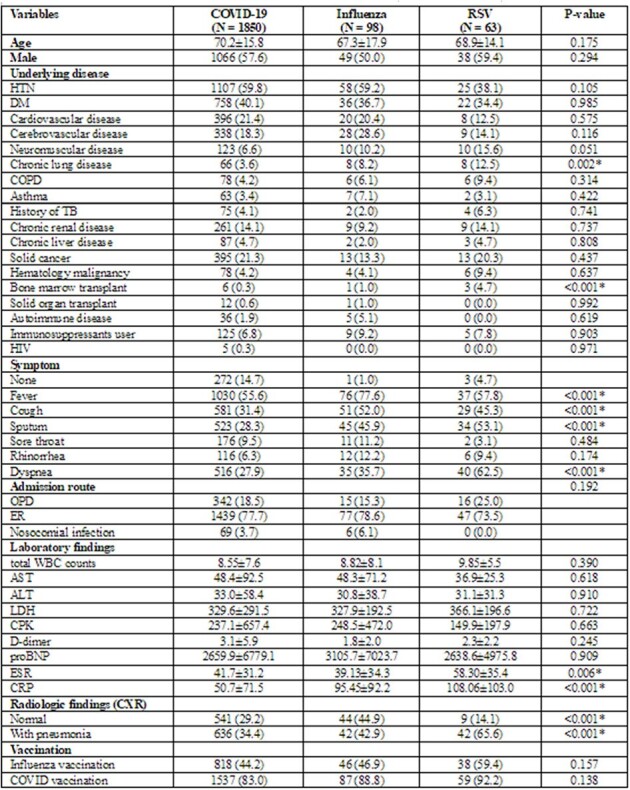
Baseline characteristics of influenza, COVID-19, and RSV. Data are presented as the mean±SD or No (%). HTN, Hypertension; DM, Diabetes Mellitus; COPD, Chronic Obstructive Pulmonary Disease; TB, Tuberculosis; HIV, Human Immunodeficiency Virus; OPD, Outpatient Department; ER, Emergency Room; WBC, White Blood Cell; AST, Aspartate Aminotransferase; ALT, Alanine Aminotransferase; LDH, Lactate Dehydrogenase; CPK, Creatine Phosphokinase; proBNP, Pro-Brain Natriuretic Peptide; ESR, Erythrocyte Sedimentation Rate; CRP, C-Reactive Protein; CXR, Chest X-Ray * P < 0.05

**Methods:**

We conducted a multi-center, retrospective cohort study utilizing Hospital-based Influenza Morbidity and Mortality (HIMM) surveillance data from 1 September 2022 to 31 August 2023. We included laboratory-confirmed cases of COVID-19, influenza and RSV among hospitalized patients aged≥19. Clinical and laboratory data were collected from electronic medical records at each participating hospital. Demographic characteristics and clinical outcomes (pneumonia, ARDS, ICU admission and mortality) were compared among three virus groups, and the risk of severe infection was evaluated by underlying chronic medical conditions.

**Table 2.**
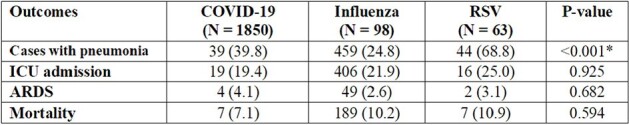
Comparison of outcomes among influenza, COVID-19, and RSV. Data are presented as the No (%). ICU, Intensive Care Unit; ARDS, Acute Respiratory Distress Syndrome * P < 0.05

**Results:**

During the study period, 1,850 laboratory-confirmed cases of COVID-19, 98 of influenza, and 63 of RSV were included in the analysis. When comparing the three virus groups, significant differences were observed in the rates of fever, cough, sputum, and dyspnea among them. Pneumonia was more common in the RSV group (65.6%) compared to the others (42.9% with influenza and 34.4% with COVID-19; p< 0.01) with significantly higher levels of erythrocyte sedimentation rate and C-reactive protein. In the COVID-19 group, the odds ratio for pneumonia increased to 1.46 (95% CI, 1.03-2.08) in the moderate-risk group with one chronic medical condition and to 1.54 (95% CI, 1.06-2.23) in the moderate-risk group with two chronic medical conditions, while showing an increase of 1.37 (95% CI, 0.90-1.93) in the high-risk group. ICU admission showed a similar trend with higher rate the moderate-risk groups. In comparison, the mortality rate was higher the high-risk group compared to the moderate-risk group (13.3% vs. 9.0%, p=0.066).

**Table 3.**
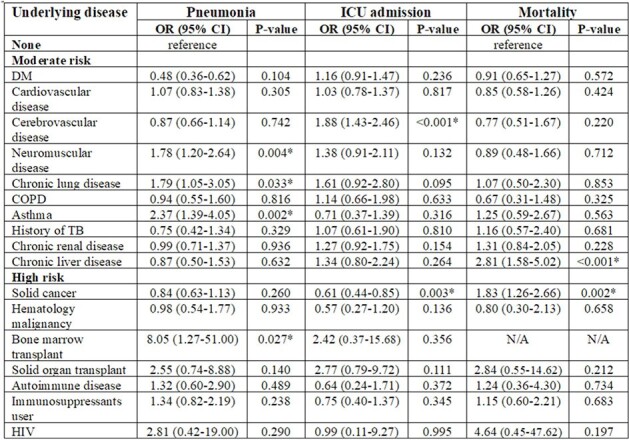
Risk assessment of pneumonia, ICU admission and mortality based on each underlying medical condition. ICU, Intensive Care Unit; OR, Odds Ratio; CI, Confidence Interval; DM, Diabetes Mellitus; COPD, Chronic Obstructive Pulmonary Disease; TB, Tuberculosis; HIV, Human Immunodeficiency Virus * P < 0.05

**Conclusion:**

Among hospitalized patients, pneumonia was more frequently identified in RSV-infected patients compared to those with COVID-19 or influenza. The risk of pneumonia development increased by underlying medical conditions.

**Table 4.**
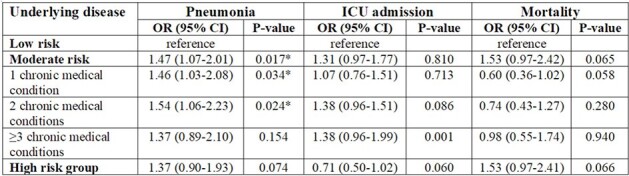
Odds ratio of pneumonia, ICU admission and mortality based on risk group. ICU, Intensive Care Unit; OR, Odds Ratio; CI, Confidence Interval * P < 0.05

**Disclosures:**

**All Authors**: No reported disclosures

